# Supplementary material for: Challenges in Assessing Repellency via the Behavioral Response by the Global Pest Tribolium castaneum to Protect Stored Grains
Source: Insects. 2024 Aug 20;15(8):626. doi: 10.3390/insects15080626 (PMC11354921; doi:10.3390/insects15080626)
Supplement: Supplementary file 1 [file insects-15-00626-s001.zip › insects-3110670-supplementary.pdf]

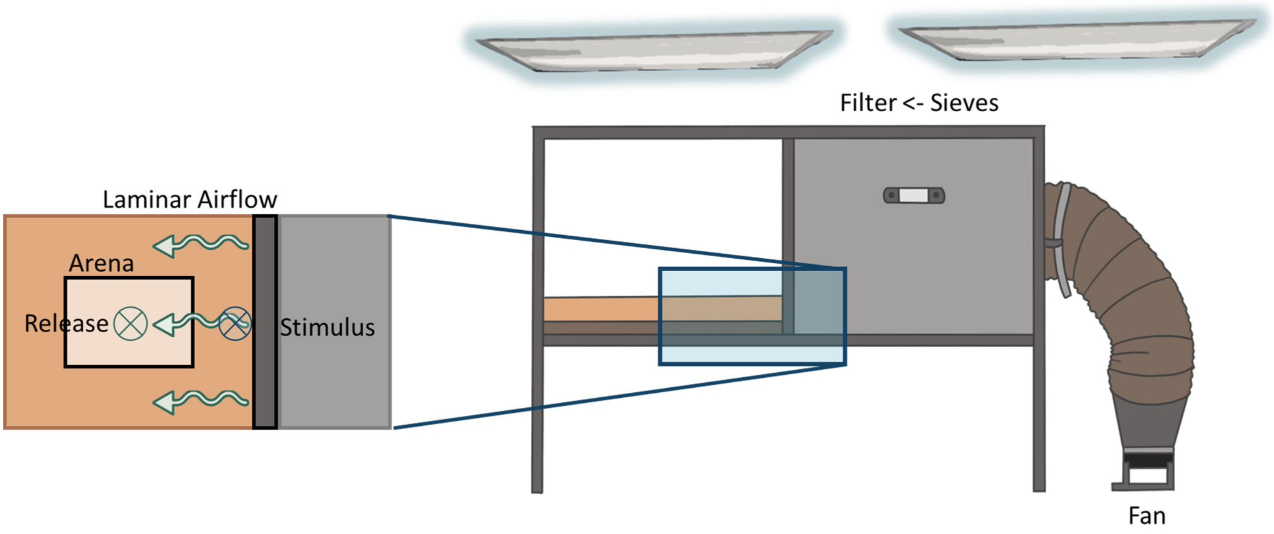

Supplementary Figure S1. Large laminar flow wind tunnel used in experimental assays. Individuals were released at the center of the arena, and then the edge on which they exited was recorded as the stimulus (side closest to the stimulus) or non-stimulus (one of the other three edges).

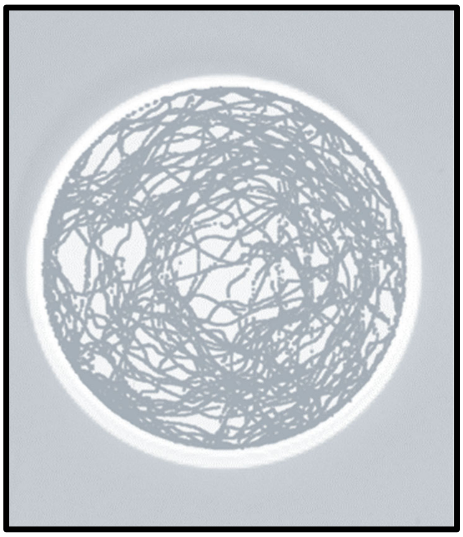

Supplementary Figure S2. Abundant and chaotic movement of 20 *T. castaneum* in response to methyl benzoquinones using Ethovision coupled with video tracking over 10 min.
